# Supplementary material for: Combined Abiotic Stresses Repress Defense and Cell Wall Metabolic Genes and Render Plants More Susceptible to Pathogen Infection
Source: Plants (Basel). 2021 Sep 18;10(9):1946. doi: 10.3390/plants10091946 (PMC8473397; doi:10.3390/plants10091946)
Supplement: Supplementary file 1 [file plants-10-01946-s001.zip › plants-1372553-supplementary/Supplementary Table S3- qRT-PCR primer sequences.pdf]

Supplementary Table S3: Real time RT-PCR primer sequences used in this study.

Primer	Sequence
PR-1 (S)	ACTACAACTACGCTGCGAACAC
PR-1 (A)	GTTACACCTCACTTTGGCACATC
PR-5 (S)	GTGTTCATCACAAGCGGCAT
PR-5 (A)	GGGAAGCACCTGGAGTCAAT
TN13 (S)	CCACCAAAACGAAAAGAGCTTG
TN13 (A)	TCGTATTCCGTTCCGTTCCG
PDF1-3 (S)	AAGACTAACTCTTCACCAGTTCAC
PDF1-3 (A)	CGGTGCTTCAAAAGCAGCAA
BOS1 (S)	CCCGTTGGGGAAATAGATGGT
BOS1 (A)	CCGAGGCTGACTGAATCCTC
THI2-2 (S)	CGAAAATTCTGGTGATGCTGTCA
THI2-2 (A)	TAGATCCTCCGGTGCAGACA
XTH20 (S)	TCCCCAAAGGGTCATTTTTACAATA
XTH20 (A)	GCGGTGAAAGGAGCTTTGGA
FLA2 (S)	CGGAGGCTTAACGGTGTTCT
FLA2 (A)	AACGACTGGTAAACCGGCAT
expG (S)	GAGCTGAAGTGGCTTCCATGAC
expG (A)	GGTCCGACATACCCATGATCC

S: Sense, A: Antisense.
